# Mesenchymal stem cells of dental origin as promising tools for neuroregeneration

**DOI:** 10.1186/scrt450

**Published:** 2014-04-29

**Authors:** Gábor Varga, Gábor Gerber

**Affiliations:** 1 Department of Oral Biology, Semmelweis University, Nagyvárad tér 4, Budapest 1089, Hungary; 2 Department of Anatomy, Histology and Embryology, Semmelweis University, Tűzoltó utca 58, Budapest 1094, Hungary

## Abstract

The adult central nervous system has only a very limited ability to newly generate lost neurons and glial cells. Therefore, its self-renewal efficiency after degenerative damage or acute injuries is very limited. Mesenchymal stem cells of various tissue origins, including dental tissues, are among the most promising tools in stem cell therapeutic approaches. In a previous issue of *Stem Cell Research & Therapy*, Ellis and colleagues demonstrated the neuronal differentiation potential of murine dental pulp stem cells. Our commentary discusses the significance of the study, the parallel efforts of other laboratories, the present limitations of neuronal transdifferentiation using cells obtained by various available methods, and the possible breakthrough by combining the various cellular resources with pharmacological and tissue engineering methods.

## 

Neurodegenerative diseases such as Alzheimer’s disease and Parkinson’s disease and traumatic disorders such as stroke or spinal cord injury are deteriorating disorders of the central nervous system. What is common in these disorders is that it is very hard, or impossible, to treat them by conventional medical therapies since the nervous system has only a very limited ability to newly generate neurons and glial cells because of the limited number of necessary precursor cells. Therefore, novel technologies such as the application of stem cells hold great promise for their cure in the near future. The present article is a commentary on original research by Ellis and colleagues [1], which appeared in a previous issue of *Stem Cell Research & Therapy* and which is of great interest in this perspective. The authors demonstrated the neuronal differentiation of dental pulp stem cells (DPSCs) from murine incisors *in vitro* and provided evidence that these cells are organized in neuronal-like networks. This is indeed just a small step toward clinical application. Yet, combined with the data of others in similar directions, it creates great hopes and high expectations that mesenchymal stem cells (MSCs) of tooth origin may serve as cellular resources to renew damaged neuronal structures.

At present, the most promising candidates among adult stem cells for regenerative therapy are MSCs, a class of multipotent cells originally identified in bone marrow. Since the discovery of MSCs in bone marrow, similar populations from other tissues have been characterized. Recent studies, including our work, have also revealed their presence in the human dental pulp and the periodontal ligament [2-4]. A very special and attractive feature of dental stem cells is that they are actually ancestors of neuronal precursors. During mammalian tooth development, the oral epithelium invaginates into the underlying neural crest-derived mesenchyme. The ectomesenchymal cells are derived from the dorsal-most aspect of the neural tube and contribute to local tissues, including the dental pulp [5]. From this aspect, it has been of great interest to identify a progenitor pool in dental tissues and investigate its regenerative potential for nervous system defects.

An increasing number of studies on human dental pulp and periodontal ligament-derived neural progenitors or neurons *in vitro* have described neuron-specific marker expression and demonstrated their functional activity [2,4,6]. These studies revealed that multi-step pharmacological transdifferentiation protocols are more efficient than simple differentiation procedures [2,4]. These studies unequivocally showed neuronal morphological differentiation and the appearance and increased expression of neuronal markers at both mRNA and protein levels. Additionally, they provided evidence for the presence of at least some functional elements that are necessary for neuronal behavior, such as specific calcium, sodium, and potassium channels. However, the functional activity of these channels showed great variability depending on the experimental settings, indicating that both the differentiation procedures and the detection methods need to be further optimized in order to receive highly reproducible transdifferentiation results with a high yield of fully differentiated neuronal cells. *In vivo*, the available information is even more sporadic. The neuroregenerative capability of MSCs of human dental pulp origin has been shown in a brain injury [7,8] and in a spinal cord injury [9] model in animals.

Although human DPSCs have strong immunoregulatory properties [10], xenotransplantation is often problematic because of immune rejection. Therefore, it is of great interest to develop rodent models for autologous or allogenic neural stem cell transplantation. Two groups, including us, reported that rat incisor DPSCs do have neurogenic potential through the successful formation of cells with neuron-like multipolar morphology that expressed neuronal markers *in vitro*[11,12], but our investigation also revealed that the efficiency of rat DPSC neurodifferentiation is much less efficient than that of human DPSCs [12]. Additionally, although the transdifferentiated rat cells showed neuronal morphology, they did not functionally exhibit the neuron-specific sodium and potassium channels (our unpublished data). This observation is actually in accordance with the observation of Ellis and colleagues [1]. They used the same protocol that we developed for human DPSCs [4] and found that neuronally differentiated murine DPSCs are immature, expressing only L-type calcium, but not neuron-specific sodium or potassium, channels. The differences in the neuronal phenotypes of human versus rodent DPSCs are certainly due in part to species differences, but the available data also suggest that the efficacy of currently available neurodifferentiation protocols has to be improved to obtain cell populations that are suitable for therapeutic purposes. Ideally, the protocols used by Ellis and colleagues [1] to transdifferentiate murine DPSCs to neuronal progenitors should be further enhanced by using the present knowledge obtained in induced pluripotent stem cell and direct reprogramming research. Despite many challenges and drawbacks, cellular therapies, including the application of MSCs of dental origin, are very promising and have great potential for curing human neuronal disorders in the future.

## Abbreviations

DPSC: Sental pulp stem cell; MSC: Mesenchymal stem cell.

## Competing interests

The authors declare that they have no competing interests.

## References

[B1] Ellis KM O’Carroll DC Lewis MD Rychkov GY Koblar SA Neurogenic potential of dental pulp stem cells isolated from murine incisors Stem Cell Res Ther 2014 5 30 10.1186/scrt419 24572146 PMC4055132

[B2] Kadar K Kiraly M Porcsalmy B Molnar B Racz GZ Blazsek J Kallo K Szabo EL Gera I Gerber G Varga G Differentiation potential of stem cells from human dental origin - promise for tissue engineering J Physiol Pharmacol 2009 60 167 175 20388961

[B3] Gronthos S Mankani M Brahim J Robey PG Shi S Postnatal human dental pulp stem cells (DPSCs) in vitro and in vivo Proc Natl Acad Sci U S A 2000 97 13625 13630 10.1073/pnas.240309797 11087820 PMC17626

[B4] Király M Porcsalmy B Pataki A Kádár K Jelitai M Molnár B Hermann P Gera I Grimm WD Ganss B Zsembery A Varga G Simultaneous PKC and cAMP activation induces differentiation of human dental pulp stem cells into functionally active neurons Neurochem Int 2009 55 323 332 10.1016/j.neuint.2009.03.017 19576521

[B5] Tucker A Sharpe P The cutting-edge of mammalian development; how the embryo makes teeth Nat Rev Genet 2004 5 499 508 15211352 10.1038/nrg1380

[B6] Arthur A Rychkov G Shi S Koblar SA Gronthos S Adult human dental pulp stem cells differentiate toward functionally active neurons under appropriate environmental cues Stem Cells 2008 26 1787 1795 10.1634/stemcells.2007-0979 18499892

[B7] Kiraly M Kadar K Horvathy DB Nardai P Racz GZ Lacza Z Varga G Gerber G Integration of neuronally predifferentiated human dental pulp stem cells into rat brain in vivo Neurochem Int 2011 59 371 381 10.1016/j.neuint.2011.01.006 21219952

[B8] Inoue T Sugiyama M Hattori H Wakita H Wakabayashi T Ueda M Stem cells from human exfoliated deciduous tooth-derived conditioned medium enhance recovery of focal cerebral ischemia in rats Tissue Eng Part A 2013 19 24 29 10.1089/ten.tea.2011.0385 22839964 PMC3530935

[B9] Sakai K Yamamoto A Matsubara K Nakamura S Naruse M Yamagata M Sakamoto K Tauchi R Wakao N Imagama S Hibi H Kadomatsu K Ishiguro N Ueda M Human dental pulp-derived stem cells promote locomotor recovery after complete transection of the rat spinal cord by multiple neuro-regenerative mechanisms J Clin Invest 2012 122 80 90 22133879 10.1172/JCI59251 PMC3248299

[B10] Li Z Jiang CM An S Cheng Q Huang YF Wang YT Gou YC Xiao L Yu WJ Wang J Immunomodulatory properties of dental tissue-derived mesenchymal stem cells Oral Dis 2014 20 25 34 10.1111/odi.12086 23463961

[B11] Sasaki R Aoki S Yamato M Uchiyama H Wada K Okano T Ogiuchi H Neurosphere generation from dental pulp of adult rat incisor Eur J Neurosci 2008 27 538 548 10.1111/j.1460-9568.2008.06026.x 18279307

[B12] Varga G Bori E Kallo K Nagy K Tarjan I Racz GZ Novel possible pharmaceutical research tools: stem cells, gene delivery and their combination Curr Pharm Des 2013 19 133 141 22950495 10.2174/13816128130118

